# ‘Deep brain stimulation is no ON/OFF-switch’: an ethnography of clinical expertise in psychiatric practice

**DOI:** 10.1007/s11097-021-09732-3

**Published:** 2021-03-09

**Authors:** Maarten van Westen, Erik Rietveld, Annemarie van Hout, Damiaan Denys

**Affiliations:** 1 Department of Psychiatry, Amsterdam UMC, University of Amsterdam, Meibergdreef 9, Amsterdam, Netherlands; 2 Research Group IT Innovations in Health Care, Windesheim University of Applied Sciences, Campus 2, Zwolle, The Netherlands

**Keywords:** Deep brain stimulation, Obsessive-compulsive disorder, Clinical expertise, Ethnography, Practice

## Abstract

Despite technological innovations, clinical expertise remains the cornerstone of psychiatry. A clinical expert does not only have general textbook knowledge, but is sensitive to what is demanded for the individual patient in a particular situation. A method that can do justice to the subjective and situation-specific nature of clinical expertise is ethnography. Effective deep brain stimulation (DBS) for obsessive-compulsive disorder (OCD) involves an interpretive, evaluative process of optimizing stimulation parameters, which makes it an interesting case to study clinical expertise. The aim of this study is to explore the role of clinical expertise through an ethnography of the particular case of DBS optimization in OCD. In line with the topic of the special issue this article is a part of, we will also use our findings to reflect on ethnography as a method to study complex phenomena like clinical expertise. This ethnography of DBS optimization is based on 18 months of participant observation and nine in-depth interviews with a team of expert clinicians who have been treating over 80 OCD patients since 2005. By repeatedly observing particular situations for an extended period of time, we found that there are recurrent patterns in the ways clinicians interact with patients. These patterns of clinical practice shape the possibilities clinicians have for making sense of DBS-induced changes in patients’ lived experience and behavior. Collective established patterns of clinical practice are dynamic and change under the influence of individual learning experiences in particular situations, opening up new possibilities and challenges. We conclude that patterns of clinical practice and particular situations are mutually constitutive. Ethnography is ideally suited to bring this relation into view thanks to its broad temporal scope and focus on the life-world. Based on our findings, we argue that clinical expertise not only implies skillful engagement with a concrete situation but also with the patterns of clinical practice that shape what is possible in this specific situation. Given this constraining and enabling role of practices, it is important to investigate them in order to find ways to improve diagnostic and therapeutic possibilities.

## Introduction

Despite technological innovations, clinical expertise remains the cornerstone of psychiatry. A clinical expert does not only have general textbook knowledge, but is sensitive to what is demanded for the individual patient in a particular situation. Signs and symptoms vary from patient to patient and require interpretation (Braude 2017). There are guidelines and scientific principles, but these need to be translated to the individual patient’s specific situation (Montgomery 2006). Precisely how the work of interpreting and translating is to be carried out cannot be fully specified (Thornton 2006). A specific research method is required to do justice to the subjective and situation-specific nature of clinical expertise.

Ethnography implies that the researcher is present in the day-to-day clinical situations where diagnostic and therapeutic activities take place, allowing him or her to observe how experts interact with the particular social and material details of these situations (Brewer 2000; Hammersley and Atkinson 2007). By repeatedly observing particular situations, the ethnographer can get a sense of the temporal dimension of activities and of relations between situations, people and materials. This focus on the life-world and its broad temporal scope makes ethnography a method particularly suited for studying clinical expertise.

An interesting case (Flyvbjerg 2006) to study clinical expertise is Deep Brain Stimulation (DBS) for Obsessive-Compulsive Disorder (OCD). Obsessive-Compulsive Disorder (OCD) is characterized by a loss of control over obsessions and compulsions (American Psychiatric Association 2013; Bürgy 2019; Stein et al. 2019). Obsessions are intrusive, persistent and anxiety-provoking thoughts about potential catastrophic consequences of one’s actions, for instance that the whole flat will burn down because you forgot to turn of the gas. Compulsions are actions that are repeatedly performed until one’s distress decreases. A patient may, for example repeatedly ask their partner to confirm that they did in fact turn of the gas. Deep Brain Stimulation (DBS) is an effective, innovative treatment for patients with severe, disabling forms of OCD that do not respond to psychotherapy or medication (Alonso et al. 2015).

What makes DBS for OCD an interesting case to study clinical expertise? DBS involves the neurosurgical implantation of electrodes in the brain and direct modulation of neurophysiology (Robbins et al. 2019; Vicheva et al. 2020). The efficacy of DBS in OCD appears to rely on clinical expertise (van Westen et al. 2019). The role of clinical expertise is especially clear in the post-surgical period of several months were clinicians optimize the DBS stimulation parameters, such as active contacts and voltage (van Westen et al. 2020). DBS optimization is an interpretive, evaluative process, that takes a considerable amount of time and effort. What makes evaluation complex is that DBS induces a broad range of changes in patients’ lived experience, as was found through qualitative interviews with OCD patients (de Haan et al. 2013; 2015; 2017). Moreover, DBS for OCD is a relatively new treatment practice that is still in full development.

The aim of this study is to explore the role of clinical expertise through an ethnography of the case of DBS optimization in OCD. In line with the topic of the special issue this article is a part of, we will also use our findings to reflect on ethnography as a method for studying complex phenomena such as clinical expertise.

## Methods

This ethnography is based on rich descriptions (Høffding and Martiny 2016) obtained through participant observation and in-depth interviews. The process of interpreting the meaning of these descriptions is structured by theoretical sampling, coding and memo-writing.

### Setting and informants

We studied a team of expert clinicians from a university hospital in the Netherlands. The team includes psychiatrists, psychologists, specialized nurses and neurosurgeons who have treated over 80 OCD patients with DBS since 2005, the largest psychiatric DBS cohort world wide. Patients are diagnosed according to DSM criteria and have, at the start of the treatment, a score on the Yale-Brown Obsessive-Compulsive Scale of at least 28 (out of 40) (Denys et al. 2020). Most patients have also comorbid disorders, with the exception of psychotic or active substance abuse disorders. Patients have a history with OCD of at least 5 years, but mostly much longer, have unsuccessfully tried multiple medications and at least one intensive course of cognitive behavioral therapy (CBT).

### Generating rich descriptions

Participant observation was performed by the first author. He was present in various clinical situations where clinicians interacted with patients and with each other (see also section “Setting, people, materials and procedures”). During sessions, the researcher wrote down observations and experiences. These field notes were written out in more detail after the session, as is common practice in ethnography (Emerson et al. 2011). Participant observations took place over extended periods of time, between October 2016 and September 2017 and between October 2018 and March 2019. These extensive observation periods made it possible to follow the entire treatment process of several patients. The gap in between both observation periods was due to other responsibilities of the first author. It had no negative implications, because the aim was not to observe everything that happened but rather to be long enough in the practice to be able to observe similarities and difference between situations. The larger total time span of observation moreover increased the chances of capturing changes in the patterns of practice.

In-depth interviews were performed by the first and second author with all clinicians who carry out the optimization of DBS stimulation parameters. This involved three specialized nurses, two psychologists and four psychiatrists. Interviews were performed by the first and second author and took 90 to 120 min. The interviews were loosely structured by a few guiding questions, including “How do you characterize an optimal DBS effect?” and “What do you do to evaluate the effects of DBS?” Next to these central questions and a flexible topic list, we encouraged clinicians to provide detailed examples, so that we could obtain rich descriptions of their lived experience at the time. Whereas there was only one real interview per clinician, participant observation provided ample possibilities for asking additional questions. Interviews were performed alongside observation so that both could mutually inform each other.

We have different degrees of affinity with the practice we are investigating. The last author is one of the members of the DBS team, the first author is a psychiatrist and the third author has been a nurse. Furthermore, the first, second and last author are philosophers and have (jointly) published about ecological-enactive cognitive science (Rietveld et al. 2018).

### Structuring the interpretive process

In order to decide for how long to continue observations and what aspects to focus on, we used theoretical sampling. Theoretical sampling implies that you let preliminary interpretations of your material and salient aspects of your previous observations guide subsequent observations (Boeije and Bleijenbergh 2019; Bowen 2006; Corbin and Strauss 2008). To minimize bias, we actively looked for so called negative cases (Boeije and Bleijenbergh 2019), exceptions that did *not* fit our preliminary interpretations. We stopped when we reached a point where new observations were foremost confirming earlier interpretations. Interpretation thus was a dialectical process in which we tried to reach a sufficient level of consistency (Høffding and Martiny 2016).

Field notes and interview transcripts were coded. This involved a first stage of open coding by the first author, in which all the material was read, divided into fragments of 2 to 10 lines, selected for relevance and assigned with a short description that captured the meaning of the fragment (Boeije and Bleijenbergh 2019). We made sure to use codes that could preserve qualitative differences. For instance, we made a general code of ‘confidence’ instead of the dichotomy ‘no confidence’ and ‘full confidence’ to capture the various degrees of confidence that clinicians encounter in interaction with patients. When thinking of a fitting description, we first consulted the growing list of codes. This also provided a means to check the existing selection of codes against new uncoded material. When a fragment did not match, a new code was created. Several interviews and part of the observation reports were also coded by the second and third authors. Differences in ways of coding choices provided an opening for discussion of how to further specify and disambiguate existing codes.

After the open coding of all material there was a second stage of axial coding where the first and third author tried out different ways in which various codes and their underlying material could be related and clustered together (Boeije and Bleijenbergh 2019). Robust and coherent clusters were given new descriptive codes and in so-called memo’s we described how these codes captured at a higher, more general level what the underlying lower-level codes were about. One example is that several lower-level codes could be linked as describing temporally related stages of a patient’s *process of improvement*. We found that clinicians’ various diagnostic and therapeutic *interventions*, which formed another cluster, could be mapped onto these stages. A third cluster captured codes about the *development of the practice*. Thus, by a process of trial and error, we found a comprehensive code structure with which we could tell a rich story about our data. The memo’s of these higher-level codes, which were built around several illustrative primary data fragments, formed an initial draft of the results section.

## Compliance with ethical standards

Based on the Research Involving Human Subjects Act, the Medical Ethics Review Committee of the Academic Medical Centre (AMC), Meibergdreef 9, Amsterdam, the Netherlands, declared that no official approval was required as our study did not subject persons to interventions or behavioral regulations. The clinicians, who were the primary participants of this study, provided verbal informed consent to being interviewed and observed. Sessions where patients were present were only attended when the patient provided verbal informed consent to the researcher’s attendance and anonymized field note taking.

## Results

For the reader to get a feel for how DBS optimization works, we first describe the setting, people, materials and procedures involved. Then we describe in general lines how clinicians make sense of DBS-induced changes in patients’ lived experience and behavior. In the final section, we zoom in on particular situations where established ways of making sense fall short and clinicians have to improvise.

### Setting, people, materials and procedures

After a neurosurgeon has implanted two electrodes into the patient’s brain and connected the implanted electrodes to a (rechargeable) battery in the chest wall there is a recovery period of three weeks. From then on a patient travels to the outpatient clinic once every two weeks for a so called “stimulation session” with a nurse or psychologist and sometimes, for part of the session, a psychiatrist. Prompted by clinicians’ questions about symptoms, side-effects and quality of life, patients tell about their daily life in the past weeks. At some point in the session clinicians ask a number of specific questions to rate several standardized questionnaires, including the 10-item Yale-Brown Obsessive-Compulsive Scale (YBOCS). The clinician gets a small hand-held device with a touch screen and a cord with a receiver and hands over the receiver to the patient who puts it against the battery. The settings of the DBS are checked and sometimes adjusted, based on an evaluation of the latest adjustment’s effects on the patient’s lived experience and behavior. This adjustment, in turn, is evaluated in the next session, and so on. Adjustments are done in a fixed order, which has been written down in a treatment protocol (van Westen et al. 2020). There are also weekly multidisciplinary team meetings with nurses, psychologists, psychiatrists and neurosurgeons. These meetings include the discussion of potential new patients, diagnostic classifications, complex cases, YBOCS scores, brain scans, troubleshooting, and planning of electrode implantation and battery-replacements. During these meetings clinicians also discuss when a patient has improved sufficiently to start with additional cognitive-behavioral therapy (CBT), which augments the effect of DBS (Mantione et al. 2014). CBT is performed by nurses or psychologists and starts when clinicians judge that stimulation parameters are sufficiently optimized. Exposure is the core of cognitive-behavioral therapy. In the context of OCD treatment, exposure implies that patients should try not to reduce the anxiety that arises as a result of obsessions through avoidance or performing compulsion. Instead, patients are encouraged to deliberately live through anxiety-provoking situations. This makes it possible to experience that the feared consequences do not occur, that compulsions may not be necessary, making it more easy to ignore obsessions.

### Attending to signs of improvement

Our analysis yielded a large variety of descriptions of DBS-related changes in patients’ lived experience and behavior as they occur at some point in the treatment. In this section we describe how clinicians *make sense* of these changes, that is, how they turn them into meaningful signs of improvement. By observing particular situations over and over, we found that there are patterns in the way clinicians make sense of DBS-induced changes. To illustrate these patterns, we will now describe how the signs of improvement that clinicians attend to become gradually more specific as a patient’s process of improvement proceeds.

When the DBS is switched on, clinicians are typically satisfied with any change that can be observed, as was illustrated in an interview with a psychologist:

So I think that during the first appointment, after it was switched on, I was happy about there being a change in the first place. I wasn’t concerned by the extent of the change or whether or not there were lots of side effects. I just wanted to see whether something would happen in that head.

Such initial changes might look like this:

You would see them curled up in their chair, morose, with a worried look in their eyes, and then suddenly, whoop! They would lift their head, they would start chatting, their voice would become livelier and their eyes light up, they’d start smiling.

If none of this occurs, the process starts all over again. Clinicians consult their protocol and adjust stimulation parameters accordingly, in the hope that this new adjustment will lead to some change.

When some initial changes have been present, clinicians shift their focus of attention to finding signs of excessive mood changes and disinhibition, which are seen as important side-effects of DBS, as was the case with the following patient:

The patient remarked that after the last increase, she had not experienced the same pleasant feeling she had before. She’s feeling on edge and restless, she can tell that she’s irritable. Her heart rate is constantly 120. She says that she has the urge to kick slow walkers ahead of her, without feeling bad about it.

Usually what clinicians initially do when such changes occur is wait for one to two weeks, as these changes are usually temporary and decrease after a couple of days. If this does not occur clinicians decrease the amount of stimulation.

After having made sure that there is at least some change and that these changes are not excessive or dangerous, there is a further specification of focus and signs of improvement become more refined. Clinicians start to look for changes in a patient’s daily routines. Often this is complicated by the fact that patients might not be aware of relevant changes, as is described by one of the clinicians, who tells about a patient who washed his hands for hours every day:

So I asked, how much time do you spend now [washing your hands]? Well, he said, 15 minutes. I said, 15 minutes, that’s not a lot at all?! I guess, he said. And then his wife chimed in – he’s a germaphobe you know – yeah, she said, I see him doing things that I haven’t seen him do in 20 years. And her husband looked at her, as if to say: What are you talking about? Well, she said, we went shopping at the supermarket and you just put the bags on the floor and put them straight on the countertop when we got home. That would’ve been entirely impossible before. But this man hadn’t realized the change at all.

Clinicians specifically look for altered daily routines, as these might indicate that patients spent less time on compulsions and that the strength of their underlying anxiety and obsessions had decreased. Note that the transcription also shows how attending to changes in the patient’s daily routines involves weighing the perspective of family-members – or nurses, when the patient is admitted in the clinic.

As changes can be very subtle and patients might themselves be unaware of them, clinicians start to direct the attention of patients themselves to changes in daily routines by saying things like:

you shouldn’t wait around on the sofa for two weeks straight, because it would be impossible to tell whether there was any improvement. You have to start doing things. Try to come up with a list of things you could do in the next 2 weeks that used to go horribly wrong but that should probably be at the lower end of things that should be achievable. Then see how much effort these activities take now and how much tension they generate. That way, we’ll have something real, something specific to discuss.

What clinicians actually do by asking patients to change their daily routines in ways that had previously been impossible, such as putting one’s shopping bags on the floor, or washing one’s hands for 15 min instead of hours, is ask patients to perform small exposure-exercises. At this point in the process, the patient’s performance in exposure-exercises primarily functions as a proxy for the effect of the DBS. Just like the patient’s family-members’ observations, these exercises increase clinicians’ sensitivity to finding changes in daily routines that might be indicative of a change in obsessions and compulsions.

Small exposure-exercises not only help clinicians in attending to subtle changes in daily routines, clinicians also use them to direct patients’ attention to these changes. What clinicians hope to achieve by this is illustrated by the following quote. A clinician compares what she is looking for in the patient to the way she deals with troubling thoughts (intrusions) herself:

I get intrusive thoughts myself as well, sometimes. It mainly happens when I’m in the car. I’ll suddenly think: oh no, I’m going to plough right into the hard shoulder. However, I’m confident enough to know that I won’t actually follow through. I know that it’s just a thought. (...) It doesn’t frighten me, because all I think is: that’s an odd thought. I would love for these patients to have the same feeling, the awareness that they won’t just give in to their thoughts.

Here we see another example of how the signs of improvement clinicians attend to become gradually more specific. At this stage of a patient’s improvement process, clinicians are attending to signs which indicate that patients have a distanced relation towards their thoughts: they can see them as “just” thoughts. Being able to adopt this stance towards one’s own thought requires patients to be aware of changes in their daily routine and to have some self-confidence in their ability to further take charge of their own thoughts and actions. Clinicians attend to these signs for a reason: increased awareness and self-confidence are important preconditions for cognitive behavioral therapy (CBT).

When clinicians finally start with full-blown CBT at the end of the optimization period, the clinicians’ signs of improvement become even more precise. It is no longer sufficient that there is some change in the patients’ daily routines. Clinicians specifically attend to finding and eradicating all remaining compulsions. Whereas adjusting stimulation parameters was initially important in moving the process forward, now this is no longer the case, as one clinician explains:

Some people (...) are able to do this all by themselves, and there’s little we have to do. When that happens, we just move smoothly to CBT. More often, we have to set a cut-off point ourselves, a clear point at which we say: let’s see whether you’re ready for part two, CBT.

By deliberately not further adjusting stimulation, clinicians create a situation in which patients can experience that they are strong enough to think ‘it’s just a thought’ and do not need the mood improvement of an increase in stimulation or the anxiety-reduction of performing a compulsion. In other words, what clinicians do by “not doing something” is move a patient’s focus of attention away from immediate satisfaction of needs towards activities that unfold over longer periods of time. These activities include CBT which is precisely about tolerating anxiety for some time to find that it decreases again. Furthermore, as compulsive routines are reduced, clinicians also try to direct the patient’s attention to healthy activities:

When we talk about confidence and security, I think that both are really fostered by an environment with more positive stimuli. I think it’s very helpful when these people start to get out of the house themselves, start volunteering, and other people start saying thank you. I really believe in that.

Thus in modulating a patient’s relation to his or her environment, clinicians are attempting to decrease the chance of the patient falling back in old compulsive routines.

### Improvisation, friction and new possibilities

In the previous section, we have described the improvement process of a typical OCD patient. In day-to-day clinical situations, however, clinicians do not encounter “typical” OCD patients. How changes in a particular patient’s lived experience and behavior are expressed might be unexpected. It might be difficult for clinicians to make sense of them in a straightforward manner, as is shown by the following observation report, which describes an interaction between a clinician and a patient:

If she were to rate her mood, she would give it a 9 out of 10. As is standard, the clinician then asks her whether there are times at which she isn’t as happy. There aren't any. This prompts the clinician to ask more detailed questions about side effects. Is she impulsive? How have her spending habits changed? Is she still sleeping? What’s she like in traffic now? Does it fit her personality? Does she get tired of herself? Or her partner? Her answers reassure the clinician that she is not at risk of potentially dangerous disinhibition. Still, the clinician mentioned later, her elated, unchanging mood continued to bother him. He linked it to his dissatisfaction with his interaction with the patient. There’s not enough reciprocity. He feels he has to do all the heavy lifting, and all his questions are met with short, succinct answers. Even in her facial expressions, the patient appears robot-like. Still, there is little reason to change the stimulation parameters, judging from how well the patient is doing in other areas. However, the clinician did decide to ask the patient to bring her partner along next time.

This description shows that although clinicians rely on their established ways of making sense, they still need to improvise. With improvisation we mean the work that is done in the particular situation to bridge the gap between, on the one hand, clinicians’ established ways of making sense and, on the other hand, the particular changes a specific patient expresses in lived experience and behavior. Is the patient in the example above currently at risk of dangerous disinhibition? That is not immediately clear. Answering this question involves interacting with the patient, through which the clinician, for instance, senses the degree of reciprocity. Interpretation also involves feelings and emotions in the clinician, such as, in this case, dissatisfaction.

Making sense of changes in the patient’s lived experience and behavior is facilitated by the clinicians’ affinity with the particular succession of changes in a typical OCD patient’s process of improvement described in the previous section. The following observation report describes how a clinician discusses with a patient how to interpret the unanticipated irritability she suffers from:

I think that changing stimulation parameters has been helpful, the clinician says. Some of your symptoms, that short fuse, that irritability, have also occurred in other patients as a side effect of DBS. In your case, though, we have in fact decreased the stimulation. A short fuse and irritability only come with overstimulation. At the same time, you’re telling me that the OCD has become more powerful, which is to be expected when the DBS is turned down. This, the clinician and the rest of the team think, must mean that the patient is suffering from symptoms that are unrelated to the DBS. What do you think of that? The patient and her partner ask whether the surgery was in vain. The clinician puts it into perspective: DBS has taught us this: borderline has to go first. (...) He recalls that a clear change occurred in the beginning of the treatment, so that DBS has had an effect. However, he says, this effect is covered by a “blanket of personality”. Before DBS can really start to help, this blanket will have to be removed first.

There are limits to improvisation. Not all changes in patients’ lived experience and behavior can be fitted into the established ways of making sense. In this example, the clinicians have first interpreted the patient’s irritability to be indicative of a certain stage of a typical OCD patient’s process of improvement. As a way of interpreting the patient’s irritability, clinicians first adopted the hypothesis that it is a side-effect of too much stimulation. This made it logical to intervene in a particular way, namely by decreasing stimulation. However, clinicians concluded that their intervention did not change the irritability and moreover worsened the OCD symptoms. This is an example of *friction* between, on the one hand, a specific patient’s lived experience and behavior and, on the other hand, clinicians’ established ways of making sense of “typical” OCD patients. Friction arises where improvisation falls short. It implies that a specific patient cannot be fitted into the established ways of making sense. Clinicians had to re-interpret the patient’s irritability as non-DBS-related and came up with an alternative interpretation of the irritability: the patient’s borderline personality disorder that had also been diagnosed at intake.

During our observations of the practice of DBS optimization, clinicians’ established ways of making sense gradually changed due to encountered friction. This was the result of a number of experiences with particular patients, including the patient discussed above, who could not be fitted into the typical process of improvement clinicians knew. These patients showed the expected initial changes in mood and in daily routines. But then these patients’ improvement stagnated. For a variety of reasons these patients had insufficient awareness and self-confidence to start CBT. During a focus group meeting, clinicians brainstormed about how to think of these patients and what to do with them:

Clinician 1 suggests that you could classify ‘non-responders’^1^ by distinguishing between purely biological/physiological effects on the one hand, and effects determined by psychological factors, personality, or insight, on the other hand. Clinician 2 responds: You should look at it as a series of doors. We’re lucky if we manage to get the patient through the first door of “DBS works”. After that, we just have to wait and see which door we’ll run into next, personality, insight, you name it. Clinician 3: Maybe, like the neurologists, we should start focusing purely on OCD and refer patients for issues related to personality. Clinician 4 advocates making the screening process stricter and to refrain from performing surgery on people with comorbid personality problems. Clinician 1 suggests to start cooperating more closely with personality treatment clinics.

Looking for ways to still accommodate these patients within the DBS treatment, clinicians decided to adopt more modest criteria for treatment success. They decided more specifically to accept the early changes in mood and daily routines as these patients’ main effect. By adopting more modest criteria for treatment success, clinicians, in fact, changed their established ways of making sense. Accepting modest criteria for success and acknowledging that they could not take away all symptoms led clinicians to start cooperating with professionals from outside the team. For some patients, clinicians started working with specialized coaches, who could help patients with structuring their daily lives. Accepting modest criteria for success also opened new possibilities. For patients with personality disorders, clinicians started cooperating with personality disorder clinics and Flexible Assertive Community Treatment (FACT) teams. As behaviors and negative affect associated with the personality disorder gradually reduced and stabilized under the influence of therapy some patients could benefit more from the DBS. Interestingly, new things became possible for these cooperating professionals as well. Now that patients had some DBS-induced improvement in mood and daily routines some patients could finally benefit from personality treatment, which had previously been impossible due to the severity of their OCD.

This was not the first instance where established patterns of making sense changed. These patterns have been established over time. Clinicians, for instance, decided to provide additional cognitive behavioral therapy (CBT) upon learning that compulsions did not sufficiently decrease with DBS alone. When CBT was thus incorporated into the treatment protocol, the signs of improvement the clinicians attend to for deciding whether or not to adjust stimulation parameters now also included the consideration whether a patient was able to start CBT treatment. Since awareness of changes is an important precondition for CBT, the introduction of CBT also put more emphasis on whether or not patients were aware of changes in their daily routines.

Another example of how previous learning experiences are reflected in the established patterns of making sense is the clinicians’ more conservative attitude towards increasing stimulation. At some point, clinicians learned that many patients ended up with too high levels of stimulation and, as a result, had side-effects such as impulsivity, irritability and sleeping problems. Moreover, they found that this had to do with the fact that many patients tended to ask rather insistently for an increase of stimulation to re-experience the mood improvement that sometimes occurs with stimulation adjustments. This is an example of how particular patterns of interaction between patients and clinicians in the past influence the amount of stimulation that is currently being applied.

Another learning experience involves clinicians’ ideas on neurobiology. The implanted electrodes have four contact points, of which some are in or near a brain area called the nucleus accumbens. This clinician describes how they decided to switch to the top two contact points to reach a different part of this brain area:

I saw that not much was happening. I was pretty disappointed. At some point, I think after about seven months or so, after the first three patients, but possibly later, I started thinking: oh dear, what if it just doesn’t really work. (...) So I thought, we’re in the bottom two contact points. We’re in the nucleus accumbens. We’re inside the core of the accumbens. And we need to get to the shell. So if I take the top two contact points, I’ll find myself in the shell of the accumbens. That was my idea, and that’s what we did. So we happened to be treating three patients at the time and saw these incredible effects. Everyone really responded amazingly. Afterwards, I took a closer look at the anatomy and saw that the shell was lower down than the core. So I hadn’t reached the shell at all. Haha. In the end, entirely flawed reasoning yielded excellent results.

This quote describes how ideas about the neurobiology, regardless of whether they are accurate, can lead to a learning experience. These learning experiences, in turn, can change clinicians’ established ways of making sense, which, as this transcription shows, have impact on how patients’ brains are stimulated.

## Discussion

Our ethnography reveals that there are patterns in the ways clinicians engage with patients. These patterns shape how clinicians make sense of changes in patients’ lived experience and behavior. These collective established patterns of clinical practice are dynamic and change under the influence of individual learning experiences in particular situations, opening up new diagnostic and therapeutic possibilities. We will now discuss what these findings imply for the practice of DBS optimization and for our understanding of clinical expertise in general and reflect on how ethnography contributes to this new understanding.

### DBS in context

In our ethnographic study, we encountered three reasons to agree with the patient who remarked that ‘DBS is no ON/OFF-switch’. First, not all effects of DBS are immediately manifest the moment the stimulation is switched on (van Westen et al. 2020). DBS stimulation parameters need to be optimized, which requires clinicians to make sense of DBS-induced changes in the patient’s lived experience and behavior. And this making sense, we found, takes time and effort.

A second way DBS is no ON/OFF-switch, our ethnographic study shows, is that DBS optimization is about more than neurophysiology alone. It is not only through adjustments in stimulation parameters that changes in the patients’ lived experience and behavior are achieved. The patient’s engagement with the life-world as a whole is optimized. The social environment of OCD patients who get a coach, for instance, is significantly restructured, opening up new possibilities for doing exposure exercises together. Inviting a patient to do exposure exercises, too, is a non-neurophysiological intervention that impacts a patients’ lived experience and behavior as well: it can make them more self-confident and aware of their improvement.

Third, DBS is no ON/OFF-switch in the sense that whether a patient will become a responder to DBS not only depends on the patient and his or her biological and/or psychological make-up. How the effects of DBS play out in a patient depends, for a large part, on the clinicians and their expertise. Since clinicians became more conservative with adjustments in stimulation parameters, for instance, less side-effects in the form of irritability and disinhibition are seen. How clinicians co-determine what ways of improvement are possible for a patient is also shown by the example that particular patients with comorbid personality problems could initially not be treated successfully for their OCD because clinicians had difficulty making sense of changes in this particular patient’s lived experience and behavior. So, how clinicians make sense has a real impact on the patient: it determines whether or not, and how, DBS parameters are adjusted – and whether patients get DBS in the first place. How the effects of DBS play out cannot be seen in isolation from the clinicians’ efforts. Decision-making, thus, involves sense-making.

We chose the term *making sense* deliberately, because we realized, as the analysis proceeded, that what can be observed with the patients and what diagnostic actions clinicians perform, are in fact two sides of the same coin. With the term *making sense* we emphasize our finding that signs of improvement are not just “out there” as stable, pre-specified entities that clinicians can readily perceive. Clinicians need to actively do something in order for changes in the patient’s lived experience and behavior to be noticed and become meaningful signs of improvement. In other words, what signs of improvement clinicians perceive depends on what they do, on how they look, on the concepts they use. Making sense implies that signs of improvement are neither completely from the patient, nor completely from the clinician. They are the product of interaction between the expressive behavior of a patient and the interpretative behavior of a clinician.

### Patterns of practice, situated interactions and ethnography

How a clinician is able to make sense of changes in a patient’s lived experience and behavior has diagnostic and therapeutic consequences. Precisely what (limited) possibilities a clinician has for making sense of a particular patient’s unique expressive behavior, this study shows, is partly determined by the established ways of making sense this clinician and his or her colleagues adhere to. The whole of established ways of making sense can be referred to as the *practice* (Nicolini 2013; Schatzki 1996; Wittgenstein 1953). A practice, it can be said, is patterned (Roepstorff et al. 2010), in the sense that it has recurrent, recognizable regularities in the ways practitioners engage with particular situations. These patterns of clinical practice can be studied, as we have done in this ethnography. Some authors argue that ethnography might also be called *praxiography* (Mol 2002). How does the practice with its established patterns of making sense relate to situations where a clinician has to make sense of a particular patient? We will first describe how the practice shapes particular situations and then we describe how, in turn, particular situations lead to changes in the practice.

Participating in the established collective patterns of a practice implies that the options for what can be done in particular situations are restricted. By adjusting stimulation parameters in a fixed order, for instance, clinicians are no longer free to pick just any combination of stimulation parameters. Yet, by restricting themselves to this collectively established order of adjusting, clinicians also make several things possible. First, knowing that their colleagues, too, stick to this fixed order helps clinicians to make sense of the story this colleague tells at a team meeting - which implies that a practice is collective. Second, in deliberately limiting their options, clinicians have created conditions to learn from experience. By adjusting the voltage in small steps in all patients, for instance, clinicians learned, at what point there was a risk of inducing side-effects. Thus, lessons from the past are carried into present encounters. The fixed order of parameter adjustments is just one of many established patterns that are constraining and enabling at the same time. Restricting their focus of attention to particular signs of improvement, for instance, enables clinicians to anticipate subtle changes that might otherwise have been missed and of which the patient him- or herself may be unaware of.

Ethnography has a dual focus on both the “zoomed-out” level of the practice and on the more “zoomed-in” level of particular situations within the practice. Because of this dual focus, ethnography is particularly suited to look at deviations relative to stable practices. Moreover, it can show how particular situations and practices are mutually constitutive (Kiverstein et al. 2019; van Dijk and Rietveld 2018). To describe this mutually constitutive relation, one may use the metaphor of ‘a path laid down in walking’ (Varela et al. 1991). A foot path through a field of high grass, for instance, shapes the actions of individuals walking on it, for it is more easy to follow the direction of the path than to make your way through high grass.

Like persons walking on a path are invited to conveniently follow it along like numerous people before them did, clinicians will interact with a new patient in ways they and their colleagues have grown familiar with through numerous other interactions with patients in the past. However, like a path through grass, established patterns of practice do not have an existence that is self-contained or independent from the situated activities of clinicians – not even when those patterns are written down in, for instance, a treatment protocol. Another way of saying this is that patterns of practice are *enacted* in particular situations (van Dijk and Rietveld^ 2017^). This implies that it is important to talk about practices always in relation to the particular situations in which clinicians keep on acting in line with the established patterns of the practice. Also the findings of this ethnography cannot be understood in isolation from the particular situated perspectives it is based on, such as those of clinicians, patients and the first author himself as participant observer.

That the practice is tied to particular situations is shown most clearly by our finding that established ways of making sense change as a result of what happens in particular situations. Should people, for whatever reason, start to walk differently, then the path’s shape will change as well. We described situations where clinicians started to act differently, thereby changing patterns of practice. Clinicians became motivated to act differently as a result of several particular situated interactions in which they feel the dissatisfaction of not being able to make sense of a patient against a background of established patterns of practice. We described situations in which clinicians encountered patients whose lived experience and behavior they could not make sense of within the interpretive framework offered by their established patterns of practice. This lived discrepancy, or *friction,* between the established patterns of the practice and the specific way a particular patient expresses his or her symptoms eventually invites exploration of alternative possibilities for action. Over time this changes the patterns of the practice and re-constitutes norms.

### Understanding clinical expertise in relation to practices

Using ethnography, we studied clinical expertise in relation to both particular situations and the practice of DBS optimization as a whole. This taught us several things about clinical expertise. In the following discussion we list five characteristics. First, we found that clinical expertise involves the ability to act in line with the established patterns of a practice. Hubert Dreyfus^2^ and other authors have rightly noted that just following the norm does not make for genuine expertise. Expertise, they take it, implies being sensitive to the demands of the particular situation and to skillfully cope with them (Dreyfus 2002; Rietveld 2008). In a previous publication on the same ethnographic study we have written in detail about this situation-specific nature of clinical judgment (van Westen et al. 2019). In the present article, too, we described situations where clinicians, in response to the particular patient they encountered, deviated from what they would normally do and had to improvise. As other authors have argued, Dreyfus (Dreyfus 2007) defines expertise too narrowly, as a completely tacit and pre-reflective set of bare behavioral reflexes. This position runs the risk of instantiating a dualism between absorbed coping, on one hand, and detached deliberation, on the other (Høffding 2014; A. Noë 2012; Rietveld 2010; Sutton et al. 2011; Thornton 2010). We studied how situated interactions relate to the patterns of the practice as a whole, which is the second characteristic of clinical expertise. Thus, we were able to paint a rich picture about clinical expertise. We showed how clinicians in the flow of their situated interactions with patients, are nevertheless still sensitive to the norms of the practice. And the practice, we showed, involves both tacit and explicit knowledge, thoughts as well as feelings.

A third characteristic of clinical expertise, which is related to the first and second characteristics, is that clinical expertise shows up in the ability to take a reflective stance towards the established patterns of the practice and how they restrict what is possible for patients and clinicians. This is what the clinicians were doing when they were brainstorming about how to deal with non-responders. Being aware of the practice in which you participate might open up ways of improving the practice. For instance, the clinicians may be moved to adopt different criteria for treatment success or to cooperate with other professionals with complementary skills. This latter example shows a fourth characteristic, namely that clinical expertise implies knowing the limits of your own skills and of the practice in which these skills are embedded.

One way of improving the practice, which might also be seen as a fifth characteristic of clinical expertise, is the ability to make creative use of the fact that practices overlap and that one is embedded in multiple practices simultaneously. Partly overlapping, other practices interfere with the practice of DBS optimization. Participating also in the practice of DBS for Parkinson Disease, for instance, helped one of the psychiatrists in the focus group brainstorm session to think of the option of adopting similar criteria for treatment success. Another example is that because the nurses and psychologists of the team also perform CBT, which is focused on eradicating as many remaining compulsions as possible, they were, even more so than the psychiatrists, focused on hearing about all the details of a patients daily routines.

The clearest example of how another overlapping practice interferes with the practice of DBS optimization is, off course, the practice of neurosurgery. How and where in the brain the electrodes are implanted co-determines how DBS is to be optimized. Patients with stimulation electrodes in the brain target area that is used in the practice we studied are more likely to experience mood improvement than patients stimulated in a different brain area (Tyagi et al. 2019). Treatment centers that use this different brain target will likely have slightly different ways of making sense, since changes in the patients are different. This shows how the brain is an integral part of the practice and also shapes the practice, as goes for the DBS device itself. Treating patients with DBS teaches clinicians new things about neurobiology, which was the case, for instance, when clinicians decided to change the contact points. In a later neuro-imaging study, they found that the contacts that had been most beneficial were located in a white matter bundle (Liebrand et al. 2019; van den Munckhof et al. 2013), which was against their initial hypothesis that DBS impacted OCD through stimulating grey matter. Implanting electrodes involves neuro-imaging. From other ethnographic studies we know that interpreting brain scans is a practice in its own right and that a great deal of interpretative work is required to render the visible images meaningful (Alač and Hutchins 2004; Mahfoud 2014; Roepstorff 2002).

### Limitations and remaining challenges

Generating rich descriptions confronted us with a dilemma. On the one hand, the richer the data we had, the more we felt able to do justice to the complexity of the phenomenon of clinical expertise. On the other hand, however, the more data we had, the more difficult it became to keep track. Coding provides some help in keeping track of large quantities of unstructured data and in finding recurrent patterns and regularities. However, analysis is more than coding alone. Not every aspect of clinical expertise could easily be captured in a coding tree. Changes in the practice unfolding over longer time-scales, for instance, do not register in the small data fragments that are usually coded. By systematically comparing particular situations in practice and by writing memo’s about these comparisons we were able to bring these changes into view.

Something we would do differently in future studies would be to code already during the periods in which we obtained rich descriptions instead of after interviews and observations were finished (Charmaz and Mitchell 2007). This could have further guided the process of theoretical sampling, possibly helping us to direct our attention to even more relevant aspects of the practice.

In the way we reported our findings in this article, we had to find a balance between, on the one hand, doing justice to the complexity of the practice and the uniqueness of each individual and each interaction and, on the other hand, the clarity and generalizability of our findings. Our focus on collective established ways of making sense downplayed the active roles of patients and materials, the ways in which each clinician is unique and how there might be different perspectives related to particular roles.

In the interviews, clinicians frequently tended to after-the-fact abstractions, to what they *think* they do instead of describing in detail what they *actually* did and experienced in a particular interaction with a patient. Including participant observation next to interviewing made it possible to observe the doings of clinicians in real-time. If we would have done a second round of interviews, we could have systematically confronted clinicians with discrepancies between what they say they do and what they actually do, which might have generated even richer descriptions of what they experience in situated interactions with patients. Another way to increase the richness of descriptions, that we would include in a future ethnographic study, is the interview techniques of microphenomenology (Petitmengin 2006). With this technique interviewers help interviewees to retrospectively re-enact their lived experience of a specific moment, by asking precise evocative questions about the spatiotemporal context and bodily sensations. This short discussion already shows that there are multiple ways of doing an ethnography, of describing a practice. Whether one uses microphenomenology, participant observation or yet other techniques is not pre-specified but rather depends on the specific practice that is being studied.

## Conclusion

This ethnography of DBS optimization sheds new light on the phenomenon of clinical expertise. By repeatedly observing situations over an extended period of time, as is central to ethnography, we found that there are recurrent patterns in the ways clinicians engage with patients. Clinical expertise should be understood in relation to these patterns of clinical practice because these patterns partly determine what is possible in particular situations. We found that participating in patterns of clinical practice helps clinicians make sense of a patient’s lived experience and behavior, to anticipate subtle signs of improvement, to decide what intervention to perform, to attune to other clinicians, to help patients become aware of their improvement, to learn from experience and to take a reflective stance. Its broad temporal scope in addition to its focus on the life-world makes ethnography particularly suited to look at deviations relative to stable patterns of practice. We found that collective established patterns of clinical practice develop under the influence of individual learning experiences in particular situations, which opens up new possibilities and challenges. An ethnographic perspective thus reveals a mutually constitutive relation between the practice as a whole and the particular situations within the practice the expert is coping with. Moreover, it is important to study patterns of clinical practice, because they have real diagnostic and therapeutic consequences.

## Footnotes

1 The official definition of response is more than 35% reduction on the YBOCS score compared to the baseline measurement before the stimulation was switched on.

2 Much research into clinical expertise in nursing studies, the only field in mainstream medical research that focuses on clinical expertise, has been inspired by the work of Dreyfus (Benner 1982; Dreyfus 2002).

## Declarations

### Availability of data and material

Data is not publicly available, but a request can be sent to the corresponding author.

## References

[CR1] Alač M, Hutchins E (2004). I see what you are saying: Action as cognition in fMRI brain mapping practice. Journal of Cognition and Culture.

[CR2] Alonso P, Cuadras D, Gabriëls L, Denys D, Goodman W, Greenberg BD, Jimenez-Ponce F, Kuhn J, Lenartz D, Mallet L, Nuttin B, Real E, Segalas C, Schuurman R, Tezenas du Montcel S, Menchon JM (2015). Deep brain stimulation for obsessive-compulsive disorder: A meta-analysis of treatment outcome and predictors of response. PLoS One.

[CR3] American Psychiatric Association (2013). *Diagnostic and Statistical Manual of Mental Disorders* (5th editio).

[CR4] Benner P (1982). From novice to expert. The American Journal of Nursing.

[CR5] Boeije H, Bleijenbergh I (2019). Analyseren in kwalitatief onderzoek: Denken en doen.

[CR6] Bowen GA (2006). Grounded theory and sensitizing concepts. International Journal of Qualitative Methods.

[CR7] Braude HD, Schramme T, Edwards S (2017). Skilled know-how, virtuosity, and expertise in clinical practice. Handbook of the philosophy of medicine.

[CR8] Brewer JD (2000). Ethnography.

[CR9] Bürgy M (2019). Phenomenology of obsessive-compulsive disorder: A methodologically structured overview. Psychopathology.

[CR10] Charmaz K, Mitchell R, Atkinson P, Coffey A, Delamont S, Lofland J, Lofland L (2007). Grounded theory in ethnography. Handbook of ethnography.

[CR11] Corbin JM, Strauss A (2008). Basics of qualitative research.

[CR12] de Haan, S., Rietveld, E., Stokhof, M., & Denys, D. (2013). The phenomenology of deep brain stimulation-induced changes in OCD: an enactive affordance-based model. *Frontiers in Human Neuroscience*, 7, 653. 10.3389/fnhum.2013.00653. 10.3389/fnhum.2013.00653 PMC3794192 24133438

[CR13] de Haan, S., Rietveld, E., Stokhof, M., & Denys, D. (2015). Effects of deep brain stimulation on the lived experience of obsessive-compulsive disorder patients: In-depth interviews with 18 Patients. *PLoS ONE*, 10(8), e0135524. 10.1371/journal.pone.0135524. 10.1371/journal.pone.0135524 PMC4552296 26312488

[CR14] de Haan, S., Rietveld, E., Stokhof, M., & Denys, D. (2017). Becoming more oneself? Changes in personality following DBS treatment for psychiatric disorders: Experiences of OCD patients and general considerations. *PLoS ONE*, 12(4), e0175748. 10.1371/journal.pone.0175748. 10.1371/journal.pone.0175748 PMC5398533 28426824

[CR15] Denys, D., Graat, I., Mocking, R., Koning, P., Vulink, N, Figee, M., Ooms, P., Mantione, M., Munckhof, P., Schuurman, R. (2020). Efficacy of Deep Brain Stimulation of the Ventral Anterior Limb of the Internal Capsule for Refractory Obsessive-Compulsive Disorder: A Clinical Cohort of 70 Patients. *American Journal of Psychiatry 177*(3):265–271. 10.1176/appi.ajp.2019.19060656 31906709

[CR16] Dreyfus H (2002). Intelligence without representation: Merleau-Ponty’s critique of mental representation the relevance of phenomenology to scientific explanation. Phenomenology and the Cognitive Sciences.

[CR17] Dreyfus H (2007). The return of the myth of the mental. Inquiry.

[CR18] Emerson RM, Fretz RI, Shaw LL (2011). Writing ethnographic Fieldnotes.

[CR19] Flyvbjerg B (2006). Five misunderstandings about case-study research. Qualitative Inquiry.

[CR20] Hammersley M, Atkinson P (2007). Ethnography: Principles in practice.

[CR21] Høffding S (2014). What is skilled coping? Experts on expertise. Journal of Consciousness Studies.

[CR22] Høffding S, Martiny K (2016). Framing a phenomenological interview: What, why and how. Phenomenology and the Cognitive Sciences.

[CR23] Kiverstein, J., van Dijk, L., & Rietveld, E. (2019). The Field and Landscape of Affordances: Koffka’s Two Environments Revisited. *Synthese*. 10.1007/s11229-019-02123-x.

[CR24] Liebrand, L. C., Caan, M. W. A., Schuurman, P. R., Van Den Munckhof, P., Figee, M., Denys, D., & Van Wingen, G. A. (2019). Individual white matter bundle trajectories are associated with deep brain stimulation response in obsessive-compulsive disorder. *Brain Stimulation, 12*(2), 353–360. 10.1016/j.brs.2018.11.014. 10.1016/j.brs.2018.11.014 30522916

[CR25] Mahfoud, T. (2014). Extending the mind: A review of ethnographies of neuroscience practice. *Frontiers in Human Neuroscience, 8, 359*. 10.3389/fnhum.2014.00359. 10.3389/fnhum.2014.00359 PMC4047819 24936177

[CR26] Mantione M, Nieman DH, Figee M, Denys D (2014). Cognitive-behavioural therapy augments the effects of deep brain stimulation in obsessive-compulsive disorder. Psychological Medicine.

[CR27] Mol A (2002). The body multiple: Ontology in medical practice.

[CR28] Montgomery, K. (2006). *How doctors think. Clinical judgment and the practice of medicine*. Oxford University Press. 10.1172/JCI33149.

[CR29] Nicolini D (2013). Practice theory, work, and organization: An introduction.

[CR30] Noë A (2012). Varieties of presence.

[CR31] Petitmengin C (2006). Describing one’s subjective experience in the second person: An interview method for the science of consciousness. Phenomenology and the Cognitive Sciences.

[CR32] Rietveld E (2008). Situated normativity: The normative aspect of embodied cognition in unreflective action. Mind.

[CR33] Rietveld E (2010). McDowell and Dreyfus on unreflective action. Inquiry.

[CR34] Rietveld, E., Denys, D., & van Westen, M. (2018). Ecological-Enactive Cognition as Engaging with a Field of Relevant Affordances: The Skilled Intentionality Framework (SIF). In A. Newen, L. de Bruin, & S. Gallagher (Eds.), *Oxford Handbook of 4E Cognition* (pp. 41–70). Oxford University Press.

[CR35] Robbins TW, Vaghi MM, Banca P (2019). Obsessive-compulsive disorder: Puzzles and prospects. Neuron.

[CR36] Roepstorff A (2002). Transforming subjects into objectivity - an “ethnography of knowledge” in a brain imaging laboratory. FOLK: Journal of Danish Ethnographic Society.

[CR37] Roepstorff A, Niewöhner J, Beck S (2010). Enculturing brains through patterned practices. Neural Networks.

[CR38] Schatzki, T. R. (1996). *Social practices. A Wittgensteinian Approach*. Cambridge: Cambridge University press. 10.16309/j.cnki.issn.1007-1776.2003.03.004.

[CR39] Stein DJ, Costa DLC, Lochner C, Miguel EC, Reddy YCJ, Shavitt RG, van den Heuvel OA, Simpson HB (2019). Obsessive–compulsive disorder. Nature Reviews Disease Primers..

[CR40] Sutton J, McIlwain D, Christensen W, Geeves A (2011). Applying intelligence to the reflexes: Embodied skills and habits between Dreyfus and Descartes. Journal of the British Society for Phenomenology.

[CR41] Thornton T (2006). Tacit knowledge as the unifying factor in evidence based medicine and clinical judgement. Philosophy Ethics and Humanities in Medicine.

[CR42] Thornton T (2010). Clinical judgement, expertise and skilled coping. Journal of Evaluation in Clinical Practice.

[CR43] Tyagi H, Apergis-Schoute AM, Akram H, Foltynie T, Limousin P, Drummond LM, Fineberg NA, Matthews K, Jahanshahi M, Robbins TW, Sahakian BJ, Zrinzo L, Hariz M, Joyce EM (2019). A randomized trial directly comparing ventral capsule and Anteromedial subthalamic nucleus stimulation in obsessive-compulsive disorder: Clinical and imaging evidence for dissociable effects. Biological Psychiatry.

[CR44] van den Munckhof, P., Bosch, D., Mantione, M., Figee, M., Denys, D., & Schuurman, P. (2013). Active stimulation site of nucleus accumbens deep brain stimulation in obsessive-compulsive disorder is localized in the ventral internal capsule. *Acta Neurochir Suppl*, 117, 53–59. 10.1007/978-3-7091-1482-7_9. 10.1007/978-3-7091-1482-7_9 23652657

[CR45] van Dijk, L. (2020). Temporalizing ontology: A case for pragmatic emergence. *Synthese.*10.1007/s11229-020-02615-1.

[CR46] van Dijk L, Rietveld E (2017). Foregrounding Sociomaterial practice in our understanding of affordances : The skilled intentionality framework. Frontiers in Psychology.

[CR47] van Dijk L, Rietveld E (2018). Situated anticipation. Synthese..

[CR48] van Westen, M., Rietveld, E., Bergfeld, I. O., de Koning, P., Vullink, N., Ooms, P., Graat, I., Liebrand, L., van den Munckhof, P., Schuurman, R., & Denys, D. (2020). Optimizing Deep Brain Stimulation Parameters in Obsessive–Compulsive Disorder. *Neuromodulation: Technology at the Neural Interface*, 24(2), 307–315. 10.1111/ner.13243. 10.1111/ner.13243 PMC7984355 33128489

[CR49] van Westen, M., Rietveld, E., & Denys, D. (2019). Effective deep brain stimulation for obsessive-compulsive disorder requires clinical expertise. *Frontiers in Psychology*, 10, 2294. 10.3389/fpsyg.2019.02294. 10.3389/fpsyg.2019.02294 PMC6817500 31695638

[CR50] Varela, F., Thompson, E., & Rosch, E. (1991). *The embodied mind: Cognitive science and human experience*. MIT Press.

[CR51] Vicheva P, Butler M, Shotbolt P (2020). Deep brain stimulation for obsessive-compulsive disorder: A systematic review of randomised controlled trials. Neuroscience and Biobehavioral Reviews.

[CR52] Wittgenstein, L. (1953). *Philosophical investigations*. Oxford: Blackwell.

